# Supplementary material for: Needs and Research Priorities for Young People with Spinal Cord Lesion or Spina Bifida and Their Caregivers: A National Survey in Switzerland within the PEPSCI Collaboration
Source: Children (Basel). 2022 Feb 27;9(3):318. doi: 10.3390/children9030318 (PMC8947533; doi:10.3390/children9030318)
Supplement: Supplementary file 1 [file children-09-00318-s001.zip › children-1580299-Supplementary.pdf]

Needs and research priorities for young people with spinal cord lesion or spina bifida and their caregivers: a national survey in Switzerland within the PEPSCI collaboration

Supplementary material B

Table S1: Age adaption of surveys

Age	Part I Basic Information	Part II PedsQL	Part III Health & Life Domain	Part IV Neurology Form	Participant information
**2-4**	p : Basic Inf. 2-14	p: PedsQL 2-4<br>yp: -	p: H&LD 2-25<br>yp: no	**Health professional (the first author)**	p: Participant Information for parents 2-10<br>yp: oral via parents/caregiver
**5-7**	p : Basic Inf. 2-14	p: PedsQL 5-7<br>yp: PedsQL 5-7	p: H&LD 2-25<br>yp: no	**Health professional (the first author)**	p: Participant Information for parents 2-10<br>yp: oral via parents/caregiver
**8-10**	p : Basic Inf. 2-14	p: PedsQL 8-12<br>yp: PedsQL 8-12	p: H&LD 2- 25<br>yp: H&LD 8-12	**Health professional (the first author)**	p: Participant Information for parents 2-10<br>yp: oral via parents/caregiver
**11-12**	p : Basic Inf. 2-14	p: PedsQL 8-12<br>yp: PedsQL 8-12	p: H&LD 2-25<br>yp: H&LD 8-12	**Health professional (the first author)**	p: Participant Information for parents 11-25<br>yp: Participant information 11-13
**13**	p : Basic Inf. 2-14	p: PedsQL 13-17<br>yp: PedsQL 13-15	p: HLD 2-25<br>yp: HLD 13-15	**Health professional (the first author)**	p: Participant information for parents 11-25<br>yp: Participant information 11-13
**14**	p : Basic Inf. 2-14	p: PedsQL 13-17<br>yp: PedsQL 13-15	p: HLD 2-25<br>yp: HLD 13-15	**Health professional (the first author)**	p: Participant information for parents 11-25<br>yp: Participant information 14-15
**15**	yp: Basic Inf. 15-25	p: PedsQL 13-17<br>yp: PedsQL 13-15	p: HLD 2-25<br>yp: HLD 13-15	**Health professional (the first author)**	p: Participant information for parents 11-25<br>yp: Participant information 14-15
**16 - 17**	yp: Basic Inf. 15-25	p: PedsQL 13-17<br>yp: PedsQL 16-17	p: HLD 2-25<br>yp: HLD 16-25	**Health professional (the first author)**	p: Participant information for parents 11-25<br>yp: Participant information 16-25
**18 - 25**	yp: Basic Inf. 15-25	p: PedsQL 18-25<br>yp: PedsQL 18-25	p: HLD 2-25<br>yp: HLD 16-25	**Health professional (the first author)**	p: Participant information parents 11-25<br>yp: Participant information 16-25

Abb: p: parents, yp: young person with Spinal Cord Injury (SCI/D) or Spina bifida (SB)

Table S2 Characteristics (age groups, gender, lesion level,) of the total number (n = 53) and for **subgroups of young people with SCI/D (n = 15) and SB (n = 38)** based on self-reports and parent proxy-reports and **SCI/D- related characteristics** (cause of injury, AIS impairment scale, lesion level, age at and time since injury)

Characteristics SCI/D and SB	Total number (%)	SCI/D number (%)	SB number (%)
**Age at survey**			
**2-4**	6 (11.3)	0	6 (15.8)
**5-7**	6 (9.4)	0	6 (15.8)
**8-12**	10 (18.9)	2 (13.3)	7 (18.4)
**13-17**	18 (34)	5 (33.3)	13 (34.2)
**18-25**	14 (26.4)	8 (53.3)	6 (15.8)
**Gender**			
**m**	26 (49)	12 (80)	14 (36.8)
**f**	27 (51)	3 (20)	24 (63.2)
**Type of injury**			
**Quadriplegia**	3 (5.7)	3 (20)	0
**Paraplegia**	43 (81.1)	12 (80)	31 (81.6)
**Not stated/ no data unavailable**	7 (13.2)	0	7 (18.4)
**SCI/D- related characteristics**			
**Cause of injury**			
**Motor vehicle/pedestrian accident**	-	3 (20)	-
**Sports/ leisure activities/Fall**	-	5 (33,3)	-
**Tumor**	-	2 (13,3)	-
**Inflammation/Infection**		1 (6,7)	-
**Bleeding**	-	2 (13,3)	
**Other accident**	-	2 (13,3)	-

ASIA			
A	-	9 (60)	-
B	-	1 (6.7)	-
C/D	-	3 (20)	-
unknown	-	2 (13.3)	-
**Lesion Level**			
C1-C3	-	0 (0)	-
C4 – C8	-	2 (13,3)	-
Th1 – Th12	-	12 (80)	-
L1 – L5	-	1 (6,7)	-
**Age characteristics**		**Mean (SD)**	
Age (Years)	-	17,9 (3.5)	-
Age at injury	-	9,9 (6.1)	-
Time since injury (Years)	-	7,5 (6.6)	-

Abb.: spinal cord injury/disorder (SCI/D), spina bifida (SB), standard deviation (SD)

Table S3: Educational level of **young people with SCI/D and SB and their parents**

Educational level	Young people SCI/D (N=15)	Young people SB (N=37)	Young people < 18 years SCI/D (N=7)	Young people < 18 years SB (N=31)	Young people > 18 years SCI/D (N=8)	Young people > 18 years SB (N=6)	Parents SCI/D (N=13)	Parents SB (N=36)
Kindergarden	0	5 (14%)	0	5 (16%)	0	0	0	0
Primary School	2 (13%)	13 (35%)	2 (29%)	13 (42%)	0	0	2 (15%)	4 (11%)
Secondary school	5 (33%)	5 (14%)	3 (42%)	5 (16%)	2 (25%)	0	1 (8%)	1 (3%)
Vocational training	4 (27%)	4 (11%)	2 (29%)	3 (10%)	2 (25%)	1 (17%)	9 (69%)	21 (58%)
University	1 (7%)	0	0	0	1 (12.5%)	0	1 (8%)	10 (28%)
Employed	2 (13%)	2 (5%)	0	0	2 (25%)	2 (33%)		
None	1 (7%)	2 (5%)	0	1 (3%)	1 (12.5%)	1 (17%)		
others	0	6 (16%)	0	4 (13%)	0	2 (33%)		

Abb.: spinal cord injury/disorder (SCI/D), spina bifida (SB)

Table S4 PedsQL™: sub scores of physical, emotional, social, and school functioning and physical health sum score (= sum of physical functioning), psychosocial health summary score (= sum of emotional, social, and school functioning), and total score: median (25 Percentile/75 Percentile) (missing) for different subgroups

Item	***All young people (n=15) SCI/D***	***All young people (n=38) SB***	***Parent-proxy reports (n=15) SCI/D***	***Parent-proxy Reports (n=38) SB***	
Physical functioning	71.9 (53.1 / 78.1) (4)	75.0 (59.4 / 87.5)(11)	57.8 (47.7 / 71.9) (3)	59.4 (40.6 / 78.1) (3)	
Emotional functioning	80.0 (60.0 / 95.0) (4)	80.0 (65.0 / 90.0) (9)	70.0 (52.5 / 85.0) (2)	60.0 (55.0 / 75.0) (2)	
Social functioning	85.0 (60.0 / 90.0) (4)	70.0 (57.5 / 82.5) (9)	80.0 (70.0 / 90.0) (1)	70.0 (56.3 / 80.0) (2)	
School functioning	81.3 (65.6 / 89.1) (5)	75.0 (62.5 / 86.3) (9)	80.0 (60.0 / 92.5) (2)	60.0 (47.5 / 70.0) (9)	
Physical Health Sum Score	**71.9 (53.1 / 78.1) (4)**	**75.0 (59.4 / 87.5)(11)**	**57.8 (47.7 / 71.9) (3)**	**59.4 (40.6 / 78.1) (3)**	
Psychosocial Health Sum Score	**84.6 (62.1 / 88.8) (4)**	**70.4 (64.2 / 84.2) (9)**	**71.7 (64.6 / 86.3) (1)**	**62.9 (55.0 / 75.0) (2)**	
Total Score	**77.4 (54.9 / 85.6) (3)**	**73.1 (62.7 / 85.2) (9)**	**70.2 (61.4 / 83.9) (1)**	**64.5 (55.0 / 72.4) (2)**	

Item	***Young people 2-4 years (n=6) SB***	***Young people 5-7 Years (n=6) SB***	***Young people 8-12 Years (n=7) SB***	***Young people 13-17 Years (n=13) SB***	***Young people 18-25 Years (n=6) SB***
Physical functioning		78.1 (68.8 / 87.5) (0)	68.8 (53.1 / 87.5) (0)	78.1 (45.3 / 81.3) (4)	65.6 (42.2 / 90.6) (1)
Emotional functioning		90.0 (68.8 /100.0)(0)	80.0 (50.0 / 85.0) (0)	75.0 (65.0 / 90.0) (2)	75.0 (65.0 / 85.0) (1)
Social functioning		87.5 (77.5 / 92.5) (0)	50.0 (40.0 / 70.0) (0)	70.0 (60.0 / 80.0) (2)	70.0 (62.5 / 90.0) (1)
School functioning		92.5 (81.3 / 96.3) (0)	62.5 (50.0 / 75.0) (0)	62.5 (56.3 / 75.0) (2)	75.0 (68.8 / 96.9) (1)
Physical Health Sum Score		**78.1 (68.8 / 87.5) (0)**	**68.8 (53.1 / 87.5) (0)**	**78.1 (45.3 / 81.3) (4)**	**65.6 (42.2 / 90.6) (1)**
Psychosocial Health Sum Score		**87.5 (78.8 / 96.3) (0)**	**64.2 (56.7 / 72.9) (0)**	**70.0 (64.2 / 76.7) (2)**	**78.3 (66.3 / 87.3) (1)**
Total Score		**85.2 (78.6 / 91.7) (0)**	**65.3 (49.5 / 78.1) (0)**	**71.4 (62.0 / 77.2) (2)**	**75.2 (60.2 / 88.1) (1)**

Item	***Parent-proxy reports 2-4 years (n=6) SB***	***Parent-proxy reports 5-7 Years (n=6) SB***	***Parent-proxy reports 8-12 Years (n=7) SB***	***Parent-proxy reports 13-17 Years (n=13) SB***	***Parent-proxy reports 18-25 Years (n=6) SB***
Physical functioning	46.9 (18.0 / 82.9) (0)	62.5 (53.9 / 68.8) (0)	65.6 (53.9 / 85.2) (1)	62.5 (31.3 / 84.4) (2)	51.6 (39.8 / 71.9) (0)
Emotional functioning	62.5 (52.5 / 75.0) (0)	57.5 (41.3 / 86.3) (0)	60.0 (55.0 / 75.0) (0)	60.0 (50.0 / 75.0) (2)	70.0 (57.5 / 88.8) (0)
Social functioning	70.0 (46.3 / 81.3) (0)	70.0 (62.5 / 76.3) (0)	60.0 (60.0 / 70.0) (0)	75.0 (55.0 / 80.0) (2)	82.5 (58.8 / 92.5) (0)
School functioning	-	60.0 (52.5 / 70.0) (0)	60.0 (50.0 / 90.0) (0)	60.0 (43.8 / 65.0) (3)	67.5 (45.0 / 82.5) (0)
Physical Health Sum Score	**46.9 (18.0 / 82.9) (0)**	**62.5 (53.9 / 68.8) (0)**	**65.6 (53.9 / 85.2) (1)**	**62.5 (31.3 / 84.4) (2)**	**51.6 (39.8 / 71.9) (0)**
Psychosocial Health Sum Score	**66.3 (49.4 / 78.1) (0)**	**60.8 (54.6 / 75.0) (0)**	**61.7 (56.7 / 68.3) (0)**	**62.5 (53.3 / 66.7) (2)**	**78.3 (55.0 / 81.7) (0)**
Total Score	**66.1 (49.8 / 82.8) (0)**	**63.2 (56.4 / 67.4) (0)**	**64.1 (56.6 / 71.6) (0)**	**61.1 (51.9 / 69.5) (2)**	**73.2 (51.8 / 81.3) (0)**

Abb.: spinal cord injury/disorder (SCI/D), spina bifida (SB)

Table S5 Health and Life Domain Questionnaire: scores for importance, satisfaction and research priority: median (missing) for young people with SCI/D and SB. The five highest ranked domains are marked in gray. The two lowest ranked domains are presented at the end of the table.

**young people with SCI/D (13–25 years)**			
	**importance**	**satisfaction**	**research**
what you do to have fun	4.54 (1)	3.77 (1)	3.15 (2)
ability to walk/wheel/move	4.46 (1)	3.46 (2)	3.08 (3)
physical functioning	4.38 (1)	3.38 (1)	3.62 (2)
relationships with friends	4.38 (1)	4.15 (1)	2.92 (2)
ability to learn new things and to concentrate	4.31 (1)	4.08 (1)	2.92 (3)
your current situation, and/or future expectations, regarding dating	4.31 (1)	3.23 (2)	2.31 (3)
time playing with or hanging out with others	4.31 (1)	3.92 (1)	2.54 (2)
fitness and exercise	4.31 (1)	3.23 (1)	3.62 (2)
relationships with family members	4.23 (1)	4.15 (1)	2.85 (2)
your school/college/university, in terms of school/college/university work	4.00 (1)	4.15 (1)	2.69 (2)
communication with others	4.15 (1)	3.92 (1)	2.92 (2)
presence of skin (pressure) sores, or ulcers, and how you can stop this from starting	3.38 (1)	3.46 (1)	3.85 (2)…
how easy it is to get where you need to go (including by car, bus, train)	4.08 (1)	3.38 (1)	3.62 (2)
ability to get around places in your community (including stores/shops, restaurants, etc.)	3.92 (1)	3.00 (1)	3.54 (2)
presence of pain and how this is treated0	3.69 (1)	3.38 (2)	3.54 (3)
ability to move your legs and feet	3.31 (2)	2.62 (2)	3.38 (3)
expectations regarding having children and being a parent	3.69 (1)	2.69 (2)	2.62 (3)
**young people with SB (13–25 years)**			
what you do to have fun	4.37 (2)	3.84 (3)	2.68 (4)
relationships with family members	4.37 (2)	4.00 (3)	2.95 (3)
relationships with friends	4.37 (2)	3.95 (3)	2.79 (3)
ability to help others	4.26 (2)	3.95 (2)	2.47 (3)
time playing with or hanging out with others	4.26 (2)	3.74 (2)	2.53 (3)
ability to get around inside your home	4.11 (2)	3.95 (2)	2.68 (3)
ability to take care of your daily personal needs (including dressing, bathing, and toileting)	4.05 (2)	3.89 (2)	3.05 (2)
presence of skin (pressure) sores, or ulcers, and how you can stop this from starting	3.79 (2)	3.16 (2)	3.58 (3)
emptying your bowel	3.95 (2)	2.89 (3)	3.53 (3)
physical functioning	4.11 (2)	3.21 (3)	3.21 (3)
how easy it is to get where you need to go (including by car, bus, train)	4.11 (2)	3.26 (2)	3.21 (2)
your equipment and assistive technologies	3.84 (2)	3.84 (2)	3.21 (3)
emptying your bladder	4.05 (2)	3.68 (3)	3.21 (3)
presence of pain and how this is treated	3.32 (3)	3.00 (3)	3.21 (4)
presence of spasms, or muscle jumping, and how you can control this	1.37 (3)	1.32 (3)	2.42 (7)
sexual activity	2.47 (2)	2.16 (3)	2.21 (7)
**parent-proxy reports SCI/D (all age groups)**			
general health	4.60(1)	2.93(1)	3.87(2)
physical functioning	4.60(1)	3.20(1)	3.67(2)
relationships with friends	4.53(1)	4.07(1)	2.27 (2)
ability to concentrate and learn new things	4.40(1)	4.13(1)	3.07(2)
accessibility of your child’s home	4.33(1)	3.27(2)	2.87(3)
communication with others	4.33(1)	4.20(1)	2.53(2)
relationships with family members	4.27(1)	4.07(1)	2.20(2)
education (school/college/university or day care), in terms of peer (social) relationships	4.13 (1)	3.93(1)	3.07(2)
ability to make him/herself heard and catch someone’s attention	4.07(1)	3.93(1)	2.60(2)
fitness and exercise	4.20(1)	3.00(1)	3.47(2)
bladder management	3.67(1)	2.47(1)	3.53(2)
presence of pain and how this is treated	4.27(1)	3.13(1)	3.47(3)
presence of spasms, or muscle jumping, and how your child can control this	3.27(1)	2.4 (1)	3.47(2)
your child’s ability to get out of his/her chair and stand	1.73(1)	1.4 (1)	2.53 (4)

support services your child receives in your home (including therapy)	2.47(2)	2,0 (2)	2.47 (3)
**parent-proxy reports SB (all age groups)**			
access to healthcare services	4.74 (1)	4.13 (2)	3.55 (3)
bowel management	4.58 (1)	3.11 (1)	4.00 (4)
bladder management	4.55 (1)	3.34 (1)	3.92 (4)
ability to concentrate and learn new things	4.50 (1)	3.45 (1)	3.61 (3)
general health	4.47 (1)	3.71 (1)	3.76 (1)
physical functioning	4.47 (1)	3.63 (1)	3.89 (2)
relationships with family members	4.47 (2)	4.37 (2)	2.61 (4)
ability to eat and drink	4.00 (1)	4.05 (1)	3.05 (5)
ability to make him/herself heard and catch someone's attention	4.37 (1)	3.97 (1)	3.13 (4)
ability to move his/her arms and hands	3.71 (1)	3.95 (1)	3.18 (4)
accessibility of your child's home	4.34 (1)	3.95 (2)	3.26 (4)
communication with others	4.34 (2)	3.95 (2)	2.63 (4)
situation, and/or future expectations, regarding employment	3.68 (1)	2.92 (2)	3.74 (3)
presence of spasms, or muscle jumping, and how your child can control this	1.37 (3)	1.63 (5)	2.61 (11)
your child's readiness, and/or future expectations, regarding having children and being a parent	2.87 (2)	2.13 (3)	3.21 (4)

Abb.: spinal cord injury/disorder (SCI/D), spina bifida (SB)

Table S6 Free text section: additional research priorities for young people with SCI/D and SB and their parents

young people with SCI/D and SB		
**Physical aspects**	**Emotional aspects**	**Social aspects**
Nerve reconstruction		Daily assistive technologies
Ability to move feet		Shopping
Perfect adapted orthesis		
Healing of spinal cord injury		
Stem cell research		
Electrostimulation of the spinal cord		
Nerves of the spinal cord		
**all parents of young people with SCI/D and SB**		
Fine motor skills	Discussion of operations not in detail in front of the child	Reconciliation of everyday life and medical best care
Gait	Increasing disrespect of people with handicaps	Child specific information regarding medical aids, barrier-free trails, inclusion
Not enough attention for bowl management.	Psychological impact of hospitalization (Fear of loss, trauma…)	
Movement of the feet	Psychological consequences of hospitalization and procedures (e.g. overhead extensions, pelvis cast)	Construction measures of schools, public transport
General anesthesia, what about side effects?		Lower costs of medical aids (wheelchairs)
Treatment of the nervous system	The ability to cope with experiences	Regulation of public toilets
Interdisciplinary cooperation	Stress management	More healthcare services should be paid by health insurance. Therapy with the Lokomat should be covered by health insurance.
Healing of spinal cord injury	Education and support of parents and caregivers, peer groups, help to cope with spinal cord injury of the child	
Muscle workout for the whole body		Less negative policy and suspicion towards sick and handicapped people
My child cannot do the bowl management on his own		
Try out more assistive technologies		Behavior of school and teachers towards handicapped children
Prevention of pressure ulcers		Full use of cognitive potential / under-challenge
Long term studies about impact of medical treatments		Coping with volume level
Impact of hydrocephalus of the development of young adults		Social integration , adapted public buildings
Reconstruction of destroyed nerves		Implement a complaints office against pro infirmis / pro cap (disability organizations)
Research about nerve cell transplant, reconstruction of nerve cells		
Better orthosis and shoes		

Abb.: spinal cord injury/disorder (SCI/D), spina bifida (SB)
